# Current status and future trends of clinical diagnoses via image-based deep learning

**DOI:** 10.7150/thno.38065

**Published:** 2019-10-12

**Authors:** Jie Xu, Kanmin Xue, Kang Zhang

**Affiliations:** 1 Beijing Institute of Ophthalmology, Beijing Tongren Eye Center, Beijing Tongren Hospital, Capital Medical University, Beijing Ophthalmology and Visual Science Key Lab, Beijing, China; 2 Royal Victorian Eye and Ear Hospital, Melbourne, Victoria 3002, Australia; 3 Faculty of Medicine, Macau University of Science and Technology, Taipa, Macau

**Keywords:** artificial intelligence, deep learning, machine learning, ophthalmology

## Abstract

With the recent developments in deep learning technologies, artificial intelligence (AI) has gradually been transformed from cutting-edge technology into practical applications. AI plays an important role in disease diagnosis and treatment, health management, drug research and development, and precision medicine. Interdisciplinary collaborations will be crucial to develop new AI algorithms for medical applications. In this paper, we review the basic workflow for building an AI model, identify publicly available databases of ocular fundus images, and summarize over 60 papers contributing to the field of AI development.

## Introduction

Artificial intelligence (AI) has recently experienced an era of explosive growth across many industries, and healthcare is no exception 1. AI will have particular utility in healthcare and will dramatically change the diagnostic and treatment pathways for many, if not most, diseases. Regardless of the specific technique, the general aim of these technologies in medicine is to use computer algorithms to uncover relevant information from data and to assist clinical decision making 2. In many developed countries and China, the application of AI technology in healthcare has developed quickly, at least in part because it enhances human resources and abilities and improves the accuracy of medical treatment. As many countries that support the development of advanced technologies welcome the incoming era of AI, they will begin to develop the necessary specifications of governance by law, regulation, technology, and standards to fully optimize this developing field of technology.

Ophthalmology is a discipline that is highly dependent on technological development. Modern ophthalmology currently makes full use of mechanical, electrical, magnetic, acoustic, optical, and other imaging technologies, and it will lead in fully implementing and adapting new technological developments such as AI. Ophthalmologists should enthusiastically embrace the development of AI technology and use it to promote advances in ocular medicine as much as possible.

## Workflow of deep learning

**Figure 1:**
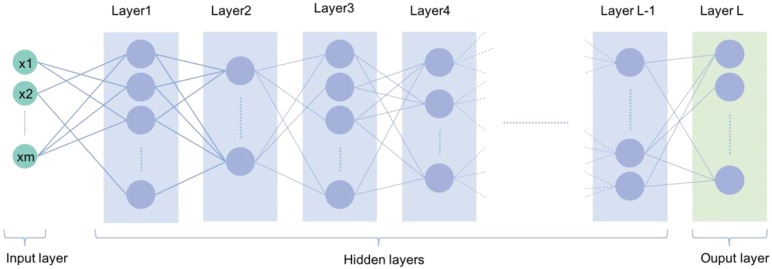
A typical deep learning neural network with multiple deep layers between input and output layers

**Figure 2:**
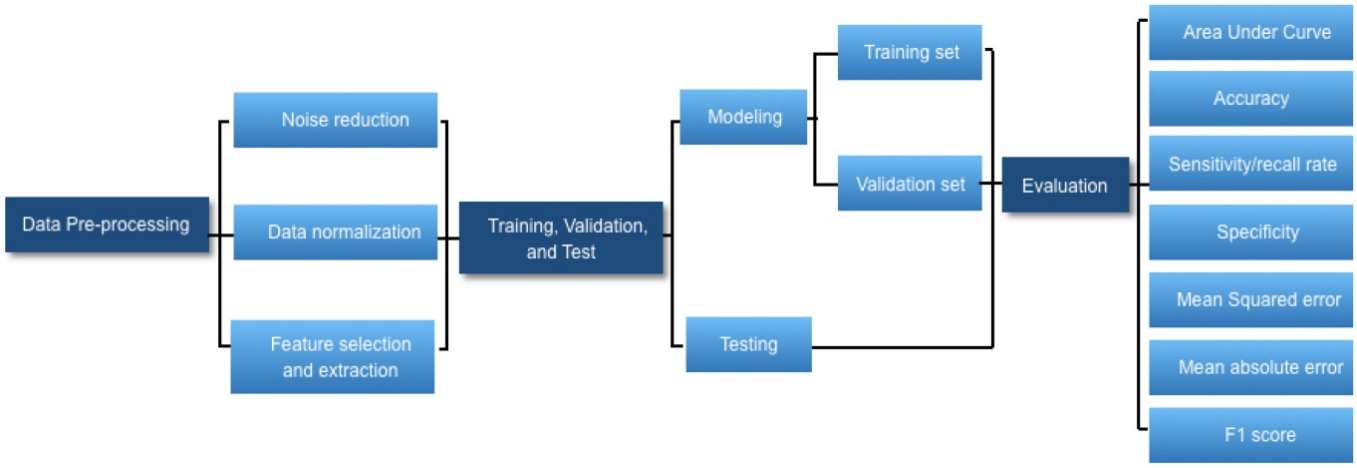
Workflow diagram of developing a deep learning-based medical diagnostic algorithm.

AI is broadly used in both the technical and popular lexicons to encompass a spectrum of learning, including but not limited to machine learning, representation learning, deep learning, and natural language processing 1. Deep learning is making major advances in solving problems that have resisted the best attempts of the AI community for many years. It is very good at discovering intricate structures in high-dimensional data and is therefore applicable to multiple medical domains 3. Deep learning discovers intricate structure in large data sets by using multiple intermediate layers positioned between the input and output layers, allowing each level to learn to transform its input signal into the following layer (Fig 1). The application of deep learning, particularly in images of the retina include classification, e.g., detection of diabetic retinopathy (DR) and diabetic macular edema (DME) in fundus photographs 4; segmentation, e.g., segmentation of the lungs 5, brain 6, cell mitosis 7; and prediction, e.g., prediction of myopia development and progression 8. The workflow of deep learning can be defined in three stages: (1) pre-processing of the image data; (2) training of the model, validation, and model testing; and (3) evaluation (Fig. 2). Data pre-processing is a critical step that is necessary to build accurate machine learning models. The pre-processed work includes noise reduction, data normalization, feature selection, and extraction 9. For training a model, we initially split the model into three sections: data training, validation, and testing. The training set enables the model to learn to fit the data parameters of the classifier. The validation set is used to prevent overfitting, and the test set is used to evaluate the performance of the trained model. Evaluation is an integral part of the development process. It helps to determine if the model will do a good job of predicting the target on new and future data.

## Common open database of retina images

Many public databases have been published, and most include instructions for use by researchers in analysis and testing. For diseases of the retina, the databases include basic pathological features that usually provide information about the seven layers of the retina and about the choroid and sclera. This type of information is compiled by a process referred to as “segmentation”, which historically has been achieved by manual image processing, but increasingly it is done by computer algorithms. This information allows comparison of the performance of different algorithms analyzing the same fundus image, with reference to the reliable implementation of a gold-standard procedure 10. Commonly used fundus databases includes DRIVE 11, STARE 12, Image-Ret 13,14, e-ophtha 15, HEI-MED 16, Retinopathy Online Challenge 17, Messidor 18, RIM-ONE 19, and DRION-DB 20. Among them, DRIVE, STARE, Image-Ret, and Messidor are used mostly to diagnose DR, while DRION-DB and RIM-ONE are used mostly for segmentation of the optic nerve head in the diagnosis of glaucoma (Table 1).

## Important research studies applying artificial intelligence to ophthalmic conditions

Image classification is a long-term research topic in the field of computer vision and pattern recognition. Recent advances of deep learning techniques have greatly facilitated the research of image classification. Many deep learning models have demonstrated performances comparable with well-trained human experts in the classification of natural images, and some have outperformed the experts. The flourish of deep learning-based image classification started with the work of AlexNet 21, where an eight-layered convolutional neural network (CNN) was designed for the classification task in ImageNet Large Scale Visual Recognition Challenge (ILSVRC). Subsequently, a series of deeper neural networks continuously refreshed the record of ILSVRC, including GoogLeNet (22 layers) 22, VGGNet (16 or 19 layers) 23, and Deep Residual Net (18, 34, 50, 101, or 152 layers) 24. These neural networks are the most widely used architectures that can achieve accurate classification for natural images by training deep models with millions of annotated images.

## Diabetic retinopathy

Diabetic retinopathy is the most common organ complication and can manifest as the earliest sign of complication of diabetes mellitus. Early detection and continuous monitoring of DR is essential to control the disease in the early stage to prevent blindness. The automatic detection of DR has attracted a lot of attention. Most automated methods use fundus images as an input. These photographs are examined for the presence of lesions indicative of DR, including microaneurysms, hemorrhages, exudates, and cotton wool spots. The application of automated image analysis to fundus images may reduce the workload and costs by minimizing the number of photographs that need to be manually graded 25. Gulshan et al 4 were the first to present a deep learning algorithm for the detection of DR in retinal fundus photographs. In 2 validation sets of 9963 images and 1748 images, at the operating point selected for high specificity, the algorithm had 90.3%and 87.0%sensitivity and 98.1%and 98.5%specificity for detecting referable diabetic retinopathy, defined as moderate or worse diabetic retinopathy or referable macular edema by the majority decision of a panel of at least 7 US board-certified ophthalmologists. Subsequently, Ting et al^26^ developed a deep learning system to detect multiple related eye diseases, including DR, possible glaucoma, and age-related macular degeneration(AMD), the Area under the receiver operating characteristic curve (AUC) of 0.936 for referable DR ,sensitivity and specificity were 90.5% and 91.6%, For vision-threatening diabetic retinopathy, AUC was 0.958,sensitivity and specificity were 100% and 91.1%. More recently, deep learning was applied to automated segmentation of optical coherence tomography (OCT) images. Kermany et al 27 developed an OCT imaging diagnostic tool based on a deep learning framework for screening patients with AMD, DME, and drusen. The classifier distinguishing DME images from normal images achieved an accuracy of 98.2%, with a sensitivity of 96.8% and specificity of 99.6%. In April 2018, the first AI diagnostic system to receive US Food and Drug Administration (FDA) approval for marketing was IDx-DR, the case of IDx-DR highlights one of the earliest successes of an AI-based technology completing the regulatory process in the United States.

## Glaucoma

Glaucoma is a group of eye diseases that damage the optic nerve and can result in irreversible vision loss and blindness and is the second leading cause of blindness worldwide. It is estimated that the disease affected 60.5 million people in 2010, and this figure is expected to reach 79.6 million by 2020 28. Currently, there is no cure for glaucoma, and vision loss, once it has occurred, is permanent. However, early detection and treatment are helpful to slow or stop the disease progression and can protect against serious vision loss. Many researchers have studied how to diagnose glaucoma automatically based on retinal images. These studies can be separated into two types. The first type outputs the glaucoma diagnosis results directly through deep learning models. Li et al 29 trained a CNN on LabelMe datasets for glaucoma diagnosis, In the validation dataset, this DL system achieved an AUC of 0.986 with sensitivity of 95.6% and specificity of 92.0%. The most common reasons for false-negative grading were glaucomatous optic neuropathy with coexisting eye conditions, including pathologic or high myopia, DR, and AMD. The leading reason for false-positive results was having other eye conditions, mainly including physiologic cupping. The second type of studies uses deep learning models to segment the glaucoma related tissues such as optic disc and optic cup, and then calculates medical measures (e.g., cup-to-disc ratio) for diagnosis. Previous studies have used various special forms of perimetry to discriminate preperimetric glaucoma from healthy eyes^30^. Asaoka et al 31 applied a DL method to differentiate the visual fields of preperimetric open-angle glaucoma patients from the healthy eyes, the AUC was 0.926.

## Age-Related Macular Degeneration

AMD is a leading cause of irreversible visual loss in the aging population; the meta-analysis conducted by Wong et al 32 suggested that AMD, was responsible for 8.7% of all global blindness. Fortunately, the anti-vascular endothelial growth factor (anti-VEGF) medications have revolutionized the treatment of exudative retinal diseases, OCT is critical to guiding the administration of anti-VEGF therapy by providing a clear cross-sectional representation of the retinal pathology in these conditions. Kermany et al 27 developed an OCT imaging diagnostic tool based on a deep learning framework for screening patients with AMD, DME, and drusen. This AI system categorized images with choroidal neovascularization and images with diabetic macular edema as “urgent referrals”, drusen as “routine referrals”, normal images were labeled for “observation”. They achieved an accuracy of 96.6%, with a sensitivity of 97.8%, a specificity of 97.4%, and a weighted error of 6.6%. The classifier distinguishing choroidal neovascularization images from normal images achieved an accuracy of 100.0%, with a sensitivity of 100.0% and specificity of 100.0%. Recently, DeepMind and the Moorfields Eye Hospital 33 developed an AI system was trained on 14 884 OCT scans to detect 9 different OCT pathologies (choroidal neovascularization, macular edema, drusen, geographic atrophy, epiretinal membrane, vitreomacular traction, full-thickness macular hole, partial thickness macular hole, and central serous retinopathy). The system was then able to recommend a referral decision based on the most urgent conditions detected, the correct referral decision with 94% accuracy, matching world-leading eye experts.

In addition to detecting and monitoring common blinding eye diseases, deep learning is also being expanded to the field of rare diseases, such as congenital cataracts and retinopathy of prematurity (ROP) in newborns. Long et al 34 constructed a CNN-based computer-aided diagnosis framework (CC-Cruiser) to classify and grade congenital cataract. In the clinical trial, CC-Cruiser achieved 98.25% accuracy with the identification networks; 100%, 92.86% and 100% accuracy for opacity areas, densities and locations, respectively, with the evaluation networks; and 92.86% accuracy with the strategist networks. Brown et al 35 reported the results of a fully automated DL system that could diagnose plus disease, the most important feature of severe ROP, for diagnosis of plus disease, the algorithm achieved a sensitivity of 93% with 94% specificity. For detection of pre-plus disease or worse, the sensitivity and specificity were 100% and 94%, respectively. In addition, retinal microvascular changes and retinopathy provide important clinical indicators for predicting the occurrence, development, therapeutic effect and prognosis of cardiovascular and cerebrovascular diseases. Poplin et al^36^ using deep-learning models trained on data from 284,335 patients and validated on two independent datasets of 12,026 and 999 patients, they predicted cardiovascular risk factors not previously thought to be present or quantifiable in retinal images, such as age (mean absolute error within 3.26 years), gender(AUC) = 0.97, smoking status (AUC = 0.71), systolic blood pressure (mean absolute error within 11.23 mmHg) and major adverse cardiac events (AUC = 0.70).Current AI studies using deep learning techniques for DR, AMD, glaucoma, cataract, and anterior ocular segment diseases are summarized in Table 2 4,26,27,29,31,34,37-63.

## Status of AI applications in clinical diagnoses

On April 2, 2019, the FDA issued a discussion paper that proposed a regulatory framework for modifications to artificial intelligence/machine learning (AI/ML)-based software as medical devices (SaMD) 64. This document explains the principles for developing artificial intelligence software, the new framework for equipment, the principles of the total product lifecycle (TPLC) certification methodology, and examples of potential real-world AI software modifications that may or may not be allowed under the proposed framework. The idea of the proposal was that with appropriately tailored regulatory oversight, AI/ML-based SaMD will deliver safe and effective software functionality that improves the quality of care that patients receive.

To date, two AI algorithms have been fully approved by the FDA and used clinically. One is IDx-DR for detecting DR, and the other is Viz.AI for analyzing images for indicators associated with a stroke. These two devices are described as “locked” algorithms, meaning that they can only be modified by the manufacturer at intervals for the purpose of “training” with new data, followed by manual verification and validation of the updated algorithm. However, there is much promise beyond locked algorithms that is ripe for health care applications. These opportunities require careful oversight to ensure that the benefits of these advanced technologies outweigh the risks to patients. These machine learning algorithms can continually evolve and are often referred to as “adaptive” or “continuously learning” algorithms. Adaptive algorithms can learn from new user data presented to the algorithm through real-world use. The FDA is exploring a framework that would allow for modifications to algorithms to be made from real-world learning and adaptation, while ensuring that the safety and effectiveness of the software as a medical device is maintained 65.

## Main challenges in the application of AI

**Figure 3:**
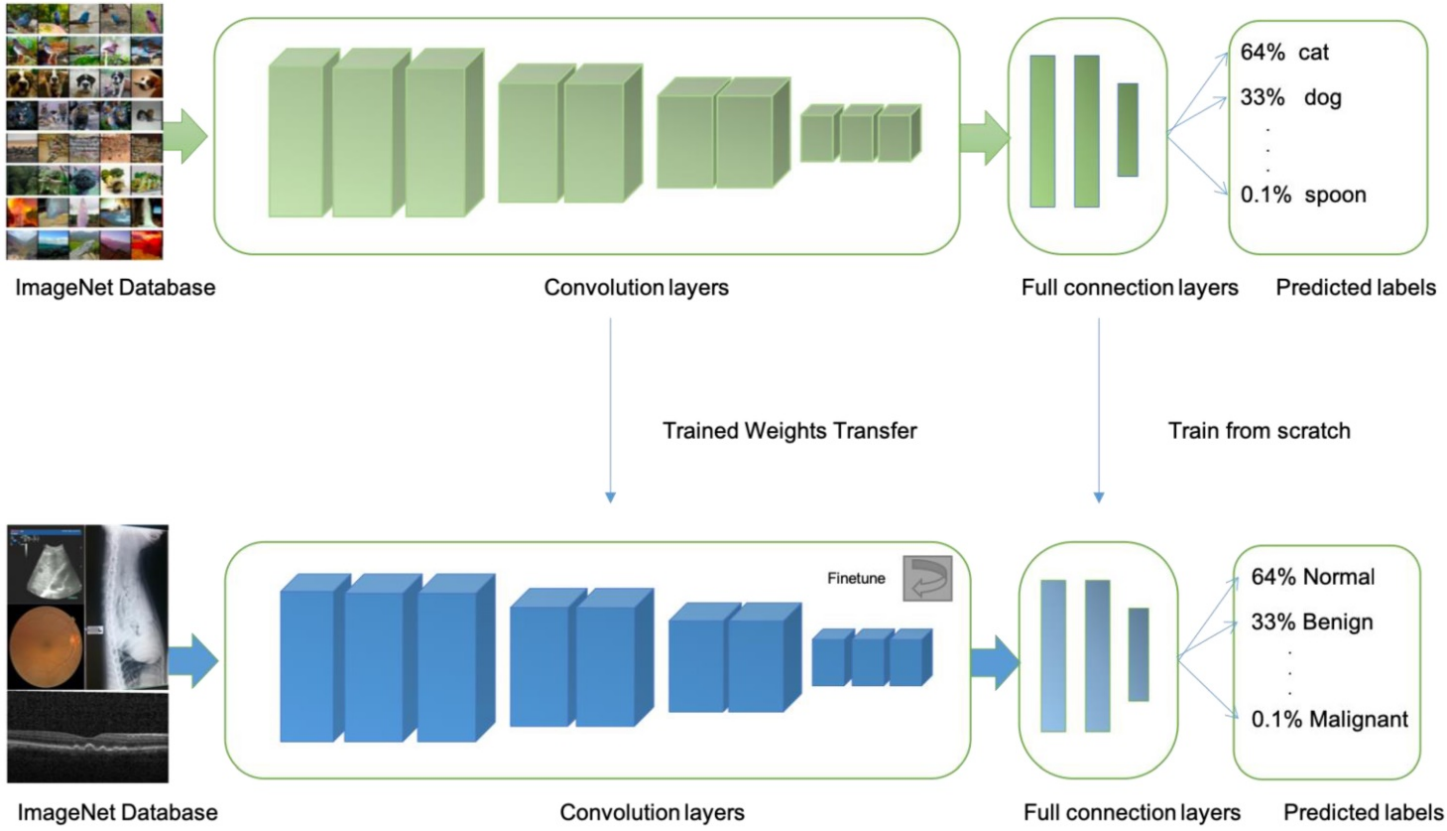
Illustrations of transfer learning: a neural network is pretrained on ImageNet and subsequently trained on retinal, OCT, X-ray images, B-scans for different disease classifications

At present, there are at least four limitations in AI technology that is based on machine learning, First, most machine learning methods have too few training sets and verification sets. More image data training is needed to further improve accuracy, sensitivity, and specificity. Transfer learning is an approach is more suitable when limited training data is available for the problem under consideration. In transfer learning, one can learn a complex model using data from a source domain where large-scale annotated images are available (e.g. natural images). Then, the model is further fine-tuned with data of the target domain where only a small number of annotated images are available (e.g. medical images) 66 (Fig 3).

The second limitation is that the examination/detection equipment used in different countries, regions, and medical institutions is not uniform; therefore, the acquired images have differences in quality and resolution, which will inevitably affect the accuracy of image analysis and thus affect the accuracy of the diagnosis. These differences will present certain obstacles in the wide-scale applications of AI technology. One solution to this problem is to unify and standardize the examination equipment. This will be difficult to achieve. Another method is to further improve AI machine learning methods at the framework and algorithm level so that they can be flexibly applied to images of different qualities while simultaneously ensuring the accuracy of intelligent diagnosis. This will increase the applicability of AI in different regions and medical institutions. However, a lot of research support is still needed in this area.

The third limitation is that the current machine learning methods for disease diagnosis lack “explanation capacity”. They do not have the ability to provide the clinician or other users with the reason for the diagnosis. The output result is based only on training and intensive learning. Thus, it is only a simple statement based upon the differences in the patient and normal images. There is no explanation for why the differences exist or the pathological basis of the differences. This, along with other issues, will affect to some extent the acceptance of these devices by doctors in clinical applications and could even provoke confusion among clinicians.

Finally, the fourth limitation in machine learning-based AI technology is that the diagnosis of some rare diseases is still unreliable. Because of the scarcity of these diseases, there are not enough cases to meet the requirements of the training and verification sets. It is difficult or impossible to ensure the accuracy of the learning model in diagnosing rare diseases. To improve the diagnosis of rare diseases, it will be necessary to optimize machine learning algorithms. This can be done by transitioning from reliance on the number of learning samples for accuracy training to utilizing combinations of various training modes and types.

## Suggestions for the application and development of AI in medicine

### 1. Consolidate the data foundation of AI applications

AI must first collect a certain amount of data in the medical industry. The structure of current medical data is very complex, in part, because there is no uniformity in the standards for compilation, and this leads to widespread information islands. It is necessary to establish a mechanism for circulating and sharing medical data. Development of data desensitization methods will promote data standardization and normalization. These methods can be used to establish standard test data sets to consolidate the data foundations of AI applications.

### 2. Define the positioning of AI development

In medicine, AI aims to help doctors (rather than substitute for doctors) to reduce the morbidity and mortality rates of patients waiting for professionals. Because doctors will not be replaced by AI, the diagnostic result of AI is only a reference for a clinical diagnosis, and doctors will always be responsible for the result. Currently, AI products in medicine play only an auxiliary role in the clinic, such as the diagnosis of DR, cancer screening, medical image recognition, disease rehabilitation, and in other fields.

### 3. Formulate regulations and laws for the application of AI in medicine

To achieve the uniform standards necessary for effective medical AI applications, laws, regulations, and other levels of governance must be established at the national level. The implementation of the national standards in industry and in routine and research clinical settings will ensure that the technology can be made widely and quickly available in the safest and most rational way. This will prudently promote the application of AI in the medical field.

### 4. Strengthen data security of AI data applications

As with the collection of any personal and medical data, the risk of inadvertent or pirated disclosure is a major concern. To reduce these risks, it is necessary to strengthen the construction of privacy protection, desensitize the data, and collect the data according to different levels and different granularities to reduce the risk of privacy disclosure. The United States has extensive experience in privacy protection of medical data. While enhancing privacy protection, it encourages the rational access and meaningful use of data and makes a selected portion of it available for research in real time, open access databases.

### 5. Promote the cultivation of interdisciplinary talents

Future development of AI in medicine can be enhanced by focusing on the integration of disciplines such as medicine, information science, and engineering. This goal can be achieved by encouraging universities, research institutes, and enterprises to cooperate with each other, set up appropriate scholarships, and establish training bases and local pilot programs. Finally, an innovative talent introduction system and mechanism should be developed to attract highly talented students to carry out innovation and entrepreneurship in the field of medical AI, thus driving the further development of the field.

## Future of AI application in clinic

Machine learning has shown its great potential in ophthalmology. Most of the current studies regarding intelligent diagnosis of eye diseases focus on dual classification problems, whereas many patients suffer from multiple categorical retinal diseases in the clinical setting. It is therefore necessary to have a model for detecting and distinguishing DR, AMD, glaucoma, and other retinal disorders simultaneously 67.

With a new generation of AI developed as a broad strategy, the applications of AI in the medical field will increase and improve. AI plays an important role in disease diagnosis and treatment, health management, drug research and development, precision medicine, etc. It can contribute significantly to solving problems of the uneven distribution of medical resources, reducing costs, and improving treatment efficiency. Applying AI helps to make up for the shortcomings of insufficient medical resources, enhance the fairness of medical services, and improve the construction of hierarchical diagnosis and treatment. In the future, AI will also offer important support for establishing an integrated medical service system. A qualified and efficient integrated medical service system can be built with the help of information-based systems.

Laws and regulations to define the legal status, responsibility sharing mechanisms, and supervision of automated systems are not yet enacted in China and most other countries. Given the complex ethical boundaries of medical AI application, the excessive control of medical AI will hinder innovation and development. On the other hand, the lack of management brings the risk of unclear subject responsibility in AI applications. Therefore, it is necessary to reasonably define AI in the medical field. The current laws on medical AI regulation are either non-existent or are in only the most primitive stages of development. There are no sound laws that regulate medical big data, the basis for medical AI. Further, there are no clear legal instructions regarding the ownership of AI data, the right to use it, privacy standards, data security, accountability norms, and whether laws can protect researchers, clinicians, and engineers from errors of innovation.

## Summary

Deep learning has the ability to probe more deeply into and discern more discriminative features from extremely large datasets. It has been applied in many research and clinical fields that rely on medical image analysis, making breakthrough progress in those disciplines. Due to unique features in ophthalmology, the diagnosis of eye diseases in clinical practice requires interpretation of many imaging studies for auxiliary diagnosis. However, detection resolution of the human eye is limited and so is human attention span. Proficiency levels of ophthalmologists also differ and it's inevitable for human errors to occur. As documented in the existing literature review, most of the current deep learning methods representing the leading level are the use of supervised learning, especially the CNN-based framework. Preliminary researches mainly focused on pre-training CNN and taking CNN as feature extractor. These pre-training networks could be downloaded directly, and conveniently applied to the analysis of any medical images. In recent two years, end-to-end training CNN has become a prioritized approach for the analysis of medical images. However, obtaining well-annotated data used for supervised learning is another major challenge for the application of deep learning to the analysis of medical images. As annotated data were usually limited at present, how to utilize unannotated images to achieve a high diagnostic accuracy using a combination of unsupervised and supervised learning will be another important development direction. In addition, electronic medical records (EMRs) contain a wealth of clinical diagnostic and treatment information that can be extracted and used to form diagnoses using natural language processing and deep learning. This information can be used to supplement the image data to formulate a complete diagnosis mimicking a human physician 68. Thus, in the near foreseeable future, AI relying on deep learning will combine image analysis with EMRs, further advancing the diagnostic power and ability to monitor disease progression and response to treatment in ways never before anticipated.

## Figures and Tables

**Table 1:** Summary of publicly available databases of ocular retinal images

Database	Number of images	Camera Model	Image Resolution (pixels)	Field of View	Application
DRIVE 11	40	Canon CR5	768×584	45°	Blood vessel segmentation
STARE 12	400	Topcon trv-50	605×700	35°	Blood vessel segmentation; Optic disk detection
Image-Ret 13,14					
DIARETDB0	130	unknown	1500×1152	50°	Diabetic retinopathy detection
DIARETDB1	89	unknown	1500×1152	50°	Diabetic retinopathy detection
e-ophtha 15					
e-ophtha_EX	82	OPHDIAT Tele-medical network	2048×1360; 1440×960	-	Diabetic retinopathy detection
e-ophtha_MA	381		1440×960; 2544×1696		Diabetic retinopathy detection
HEI-MED 16	169	Zeiss Visucam PRO fundus camera	2196×1958	45°	Hard exudate detection; Diabetic macular edema assessment
Retinopathy Online Challenge 17	100	Canon CR5-45-NM	768×576; 1058×061; 1389×1383	45°	Microaneurysms detection
Messidor 18	1200	TopCon TRC NW6	1440×960; 2240×1488; 2304×1536	45°	Diabetic retinopathy detection
RIM-ONE 19	169	Nidek AFC-210 with a body of a Canon EOS 5D	2144 × 1424	-	Optic nerve head segmentation
DRIONS-DB 20	110	Color analogue fundus camera digitized by HP-PhotoSmart-S20 scanner	600×400	-	Optic nerve head segmentation

**Table 2:** Summary of influential papers on ophthalmic image analysis

Disease	Procedures / examinations	Data sets	Deep learning techniques	Performance	Reference	

Keratoconus	Pentacam	194 normal, 454 keratoconus, 67 forme fruste, 28 astigmatic, 117 after refractive surgery	SVM	Acc: 98.9%; Sen: 99.1%; Spe: 98.5%; AUC:0.99	Hidalgo et al. 37	
	Dual Scheimpflug Analyzer	177 normal, 148 keratoconus	Decision Tree	Sen: 100%; Spe: 99.5%	Smadja et al. 38	
	Pentacam HR	30 normal, 15 unilateral keratoconus, 30 bilateral keratoconus	FNN	Bilateral keratoconus versus normal; AUC: 0.99; Sen: 100%; Spe: 95%	Kovacs et al. 39	
Pterygium	Anterior segment photographed images	2,692 non-pterygium, 325 pterygium	Shape features + SVM/ANN	Acc: 91.27%; AUC: 0.956; Sen: 88.7%; Spe: 88.3%	Zaki et al. 40	
Cataract	Slit-lamp image	5,378 images with decimal grading scores ranging from 0.1 to 5.0.	CRNN	70.7% exact integral agreement ratio (R0); 88.4% decimal grading error ≤ 0.5 (Re0.5); 99.0% decimal grading error ≤ 1.0 (Re1.0 ).	Gao et al. 41	
	Slit-lamp image	476 normal, 410 cataract	DCNN	Cataract vs Normal: Acc:98.87%;Sen:98.78% Spe:98.95%	Long et al. 34	
	Fundus image	767 normal, 472 cataract (246 mild cataract,128 moderate cataract, and 98 severe cataract)	SVM, BPNN	Acc: 93.2% for detection,84.5% for grading; Sen:94.2% for detection,74.6-89.3% for grading; Spe:91.5% for detection,90.4-98.9% for grading	Yang et al. 42	
	Slit-lamp image	476 normal, 410 pediatric cataract	CNN, SVM	Acc, Sen, and Spe: classification (97.07%, 97.28%, and 96.83%,) three-degree grading area (89.02%, 86.63%, and 90.75%) density (92.68%, 91.05%, and 93.94%) location (89.28%, 82.70%, and 93.08%)	Liu et al. 43	


POAG	Fundus image	Training set:125,189; Validation set: 71,896	DLS	AUC: 0.942; Sen: 96.4%; Spe: 87.2%	Ting et al. 26	
	Fundus image	Training set: 31,745; Validation set: 8,000	DCNN	AUC: 0.98; Acc: 92.9%; Sen: 95.6%; Spe: 92.0%; AUC: 0.986	Li et al. 29	
	Fundus image	589 normal, 837 glaucoma	CNN	Acc: 98.13%; Sen: 98%; Spe: 98.3%	Raghavendra et al. 44	
	Fundus image	30 normal, 30 open-angle glaucoma	SVW	Acc:91.67; Sen:90%;Spe:93.33%	Krishnan et al. 45	
	Visual field	Training set:257; Test set: 129	ANN	AUC: 0.890; Sen: 78.3%; Spe: 89.5%	Oh et al. 46	
	Fundus image	266 normal, 72 mild, 86 moderate, 86 severe glaucoma	SVM	Acc: 93.1%; Sen: 89.75%; Spe: 96.2%	Acharya et al. 47	
	Fundus image and SLO image	Normal/glaucoma Fundus images:85/39; Normal/glaucoma SLO images: 46/19	RIFM	Acc for Fundus images: 94.4%,SLO images: 93.9%;Sen for Fundus images: 92.3%,SLO images: 89.5%;Spe for Fundus images: 95.3%,SLO images: 93.5%	Haleem et al. 48	

	Visual fields	53 glaucoma eyes, 108 normal eyes	FNN	AUC: 92.6%, The sensitivity was 77.8%,54.6%, and 50.0%, respectively, at the specificity of 90%, 95%,and 99%	Asaoka et al. 31	
DR	Fundus image	Training set:76,370; Validation set: 112,648	DLS	For referable DR: AUC: 0.936; Sen: 90.5; Spe: 91.6%; For vision-threatening DR: AUC: 0.958; Sen: 100%; Spe: 91.1%	Ting et al. 26	
	Fundus image	Development Data Set (EyePACS in the United States and 3 eye hospitals in India): 128,175 Validation Data Set (EyePACS-1: 9,963; Messidor-2: 1,748)	DCNN	AUC: 0.991 for EyePACS,0.990 for Messidor;Sen: 90.3% for EyePACS,87% for Messidor;Spe: 98.1% for EyePACS,98.5% for Messidor	Gulshan et al. 4	
	Fundus image	170 DR, 170 normal	PNN-GA, SVM quadratic kernels	PNN-GA: Acc:99.41%,Sen:99.41% Spe:99.41%; SVM: Acc:99.12 % Sen:98.82%,Spe:99.41%	Ganesan et al. 49	
	Fundus image	EyePACS: 75,137 DR images; External validation: MESSIDOR 2 and E-Ophtha	DCNN	AUC 0.94 for Messidor 2, 0.95 for E-Ophtha;Sen 93% for Messidor 2,87% for E-Ophtha;Spe 90% for Messidor 2,94% for E-Ophtha	Gargeya et al. 50	
	Fundus image	Training set: 327 diabetic patients; Validation set: 725 diabetic patients	LASSO	Acc: 89.2%; AUC: 0.90; Sen: 75%; Spe: 89.6%	Oh et al. 51	
	Fundus image	Training set: 400; Testing set: 9,954	Ensemble of classifiers with hidden Markov chain for context information, trained by genetic algorithm	Sen: 92.2%; Spe: 90.4%	Tang et al. 52	
	Fundus image	Messidor-2 dataset: 1,748	CNN	Referable DR: AUC: 0.980; Sen: 96.8%; Spe: 87%; Vision threatening DR: AUC: 0.989; Sen: 100%; Spe: 90.8%	Abramoff et al. 53	
	Fundus image	4,445 DR; 5,494 normal	DCNN	Acc: 0.81	Takahashi et al. 54	
	Fundus image	DIARETDB1, FAZ, MESSIDOR, Private dataset: 750 (Normal: 150, mild NPDR: 150, moderate NPDR: 150, severe NPDR: 150, PDR: 150)	DNN	AUC: 0.924; Sen: 92.18%; Spe: 94.50%	Abbas et al. 55	
DME	SD-OCT	Training set: 11,349; DEM; 51,140 normal; Validation set: 250 DME, 250 normal	CNN	Acc: 98.2%; Sen: 96.8%; Spe: 99.6%	Kermany et al. 27	
DME	Fundus image	283 DR; 1,086 normal	Ensemble of Gaussian mixture model and SVM with RBF kernel	Acc: 96.8%; Sen: 97.3%; Spe: 95.9%	Akram et al. 56	


AMD	Fundus image	Training set 72,610; Validation set: 35,948	DLS	AUC: 0.931; Sen: 93.2%; Spe: 88.7%	Ting et al. 26	
	Fundus image	AREDS dataset: >130,000	DCNN	AUC: 0.94∼0.96 Acc: 88.4%∼91.6% Sen: 71%∼88.4% Spe: 91.4%∼94.1%	Burlina et al. 57	
	Fundus image	AREDS dataset: 5,664	DCNN	Acc 79.4% (4-class) 81.5% (3-class); 93.4% (2-class)	Burlina et al. 58	
	SD-OCT	Training and validation sets: 1,012 (AMD: 701; normal: 311); Test:100 (AMD: 50, normal: 50)	DCNN	Acc: 96%; Sen: 100%; Spe: 92%	Treder et al. 59	
	Fundus image	135 AMD subjects, 135 normal subjects	Feature extracted by Discrete wavelet transform and others for feature selection and classification	Average Acc: 93.7%; Sen: 91.11%; Spe: 96.3%	Mookiah et al. 60	


	OCT	48,312 AMD; 52,690 normal	DCNN	AUC: 0.975; Sen: 92.6%; Spe: 93.7%	Lee et al. 61	
	SD-OCT	1,289	CNN	The mean Dice coefficient for human interrater reliability and deep learning were 0.750 and 0.729, respectively.	Lee et al. 62	
CNV	SD-OCT	Training set: 37,206 CNV, 51,140 normal; Validation set: 250 CNV, 250 normal	CNN	Acc: 100%; Sen:100%; Spe:100%	Kermany et al. 27	
CNV	Fluorescein angiography	33	AdaBoost	Accuracy: 83.26%	Tsai et al. 63	

RBFNN, radial basis function neural network; SVM, support vector machine; MLP, multi-layer perceptron; CRNN, convolutional-recursive neural networks; DCNN, Deep-learning convolutional neural network; BPNN, Back propagation neural network; DLS, deep learning system; CNN-FE, convolutional neural networks feature-exaggerated; MLP-BP, Multilayer Perceptron with Back Propagation; RIFM, Regional Image Features Model; FNN, feed-forward neural network; PNN-GA, probabilistic neural network-genetic algorithms; LASSO, least absolute shrinkage and selection operator; NB, naive Bayes; PNN, probabilistic neural network; RBF, Radial basis function; SD-OCT, spectral domain optical coherence tomography; SLO, Scanning Laser Ophthalmoscopy. Acc, accuracy; Sen, sensitivity; Spe, specificity; Vs, versus; AUC, area under the curve; POAG, primary open-angle glaucoma; AMD, age-related macular degeneration; OCT, optical coherence tomography; DR, diabetic retinopathy.
